# Pneumococcal-meningitis associated acute disseminated encephalomyelitis (ADEM) – case report of effective early immunotherapy

**DOI:** 10.1186/2193-1801-3-415

**Published:** 2014-08-08

**Authors:** Konstantin Huhn, De-Hyung Lee, Ralf A Linker, Stephan Kloska, Hagen B Huttner

**Affiliations:** Department of Neurology, University Hospital Erlangen, Schwabachanlage 6, 91054 Erlangen, Germany; Department of Neuroradiology, University Hospital Erlangen, Erlangen, Germany

**Keywords:** Acute disseminated encephalomyelitis, ADEM, Bacterial meningitis, Streptococcus pneumonia, Parainfectious disease

## Abstract

**Introduction:**

Unvaccinated patients with history of splenectomy are prone to fulminant courses of Streptococcus pneumoniae-associated bacterial meningitis. Besides direct brain damage those patients may additionally suffer from parainfectious syndromes, notably vasculitis and acute disseminated encephalomyelitis (ADEM). Differentiation and treatment of these immunological reactions is challenging.

**Methods:**

Case report.

**Results:**

A 61 year-old woman with history of splenectomy without vaccination for S. pneumoniae presented with progressive headache and meningism. CSF-analysis revealed pleocytosis with microbiological evidence for pneumococcal meningitis. After unsuspicious initial cranial CT imaging and initiation of appropriate antibiotic therapy, MRI two days later showed widespread FLAIR- and T2-hyperintense white matter lesions that further progressed upon follow-up MRI and that fulfilled imaging criteria of ADEM. Meanwhile the patient deteriorated and required mechanical ventilation. Cranial angiography showed no signs of vasculitis or vasospasms. Screening for autoimmune diseases remained negative, however oligoclonal bands turned positive. Brain biopsy mainly revealed perivascular CD4+ T-cells and demyelinated areas. Despite ongoing acute meningitis, a 10-day corticosteroid-pulse was initiated followed by steroid-tapering. Within 4 weeks, clinical and MRI findings ameliorated. In an one-year follow-up visit, the patient significantly recovered, MRI lesions were markedly reduced and no further relapses occurred.

**Conclusion:**

Acute pneumococcal meningitis in unvaccinated splenectomized patients may be complicated by a monophasic course of parainfectious ADEM that can be controlled with high-dose corticosteroids. Parainfectious vasculitis or cerebritis are important differential diagnoses and exact differentiation of these entities is important to initiate early appropriate immunotherapy.

## Introduction

Bacterial meningitis in adults is most commonly caused by Streptococcus pneumoniae and often shows a fulminant clinical course (Weisfelt et al. 2006). Especially patients with a history of splenectomy – without vaccination – are at risk of an accompanying “overwhelming post-splenectomy infection” (OPSI) (Morgan and Tomich 2012). Besides direct brain damage due to cerebral infection, there is some evidence that patients with bacterial meningitis may additionally suffer from parainfectious inflammatory syndromes such as vasculitis or acute disseminated encephalomyelitis (ADEM) (Ohnishi et al. 2007)^,^ (Beleza et al. 2008; Okada and Yoshioka 2010) which is usually more common in younger patients (Tenembaum et al. 2007). However, an exact differentiation of these immunological reactions is often challenging.

## Case report

**Figure 1:**
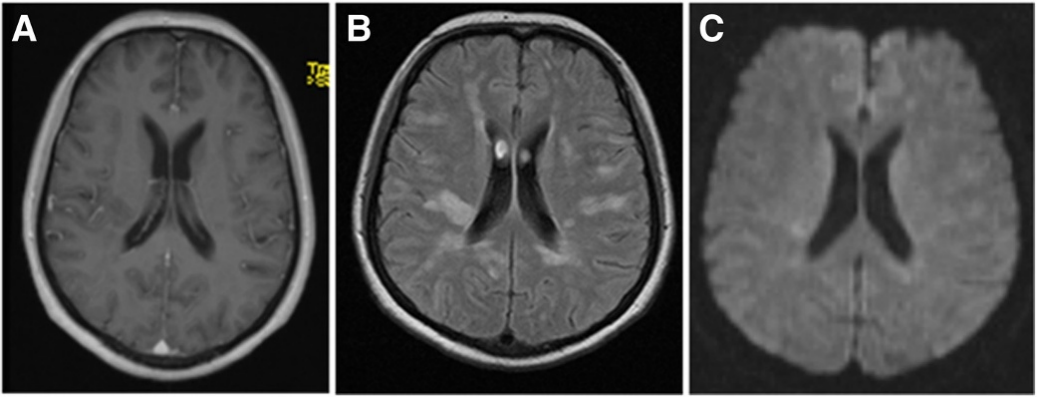
**MRI-scan two days after symptom onset.** Emerging white matter lesions with slight restricted diffusion but without Gadolinium (Gd) enhancement. **A)** T1 Gd-enhanced, **B)** T2-FLAIR (fluid attenuated inversion recovery), **C)** DWI (diffusion-weighted imaging).

**Figure 2:**
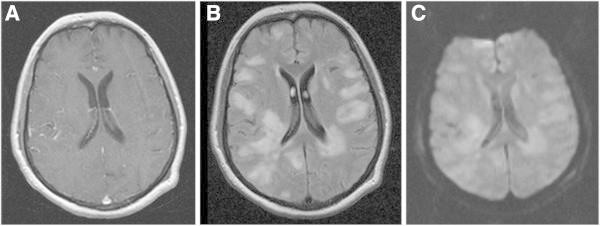
**MRI-scan 10 days after symptom onset.** Progressive widespread white matter lesions with restriction in diffusion but still unaffected blood-brain-barrier. **A)** T1 Gd-enhanced, **B)** T2-FLAIR, **C)** DWI.

**Figure 3:**
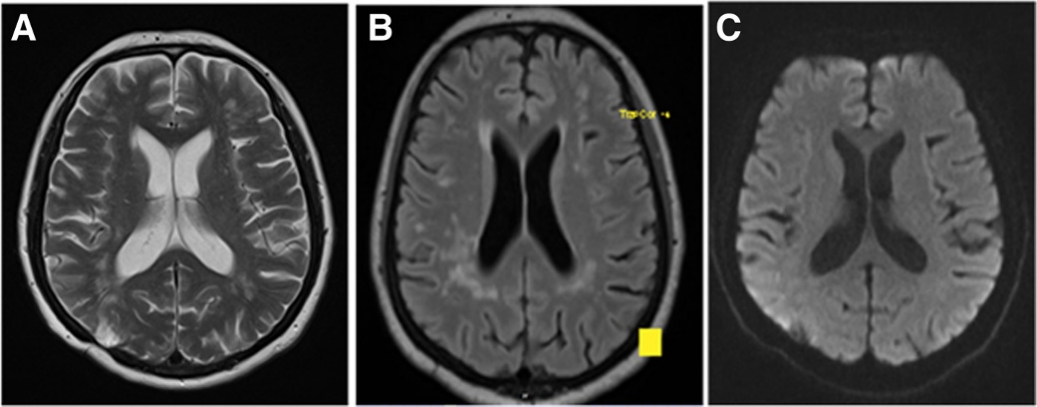
**MRI-scan at 1-year follow-up visit.** Markedly reduced white matter lesions. Right occipital hyperintensity on T2 reflects brain biopsy area. **A)** T2, **B)** T2-FLAIR, **C)** DWI.

A 61 year-old woman with a history of splenectomy in childhood - without any vaccination for Streptococcus pneumoniae - presented in our emergency room with reduced general condition, subfebrile body temperature, progressive headache, bilateral hypacusis and signs of meningism. Laboratory values for systemic inflammation were markedly elevated (16.900 leukocytes/μl, CRP 420 mg/dl, procalcitonin 7.9 μg/l) and CSF-analysis revealed neutrophil dominated pleocytosis of 98 leukocytes/μl (2 days later: 5.200 cells/μl) as well as highly elevated lactate (22.9 mmol/l) and protein (5.8 g/l), whereas glucose was barely measurable. Initial cranial CT imaging was normal and calculated initial therapy consisting of ceftriaxon, ampicillin, aciclovir and low dose dexamethasone was started immediately (de Gans and van de Beek 2002). After microbiological confirmation of Streptococcus pneumonia in CSF and blood cultures antibiotic treatment was switched to penicillin G according to resistance screening.Two days after symptom onset the patient clinically deteriorated, showed a progressive tetraparesis and decreased vigilance requiring mechanical ventilation. MR imaging of the neuroaxis revealed FLAIR- and T2-hyperintense white matter lesions without gadolinium enhancement and no restrictions in diffusion-weighted sequences (Figure 1), whereas spinal cord was unaffected. Despite multidisciplinary investigation no additional source of infection was found. Serological screening for autoimmune-mediated and paraneoplastic diseases including ANA, ANCA, Anti-dsDNA, IL-2 receptor, ACE, CCP, Anti-Cardiolipin, beta2-microglobulin, AMA, Anti-histone, Anti-nucleosome, Anti-PCNA, Anti-centromer B, Anti-Jo/-Hu/-Ri, Anti-SS-A/B, Anti-Scl70, Anti-Ro52, Anti-PM-Scl, Anti-RNP-A/-C/-70, Anti-Sm, Anti-RNP/Sm, kappa/lambda light-chains, C3/4 complement, IgA/G/M was negative. Moreover, metabolic disorders were excluded and virological testing for herpes viral (HSV, VZV, CMV, EBV), influenza, picorna, HIV and JCV infection was unremarkable.Follow-up MRI after 10 days – while the patient remained comatose during interim sedation pauses – revealed a progression of the bilateral white matter lesions and a new slight gadolinium (Gd) enhancement (Figure 2) and MR-spectroscopy showed a reduced N-acetyl-aspartate (NAA) peak. At this time-point CSF cell count normalized (4 cells/μl, predominantly lymphocytes), however oligoclonal bands had turned positive. Digital substraction angiography was negative for signs of cerebral vasculitis, septic central venous thrombosis or vasospasms. Electrophysiological investigation indicated critical-illness polyneuropathy (CIP) and EEG showed signs of a diffuse encephalopathy without epileptogenic potentials. For proper diagnosis of the leukencephalopathy and to rule out cerebritis or abscess, brain biopsy of a large white matter lesion was performed which mainly revealed perivascular CD4+ T-cells with signs of demyelination but without pathological features for vasculitis or neoplasia. PCR from biopsy material could not detect any viral or bacterial pathogen.Assuming an accompanying parainfectious inflammatory process - most likely ADEM -, corticosteroid treatment was initiated despite ongoing active bacterial meningitis (CRP > 200 mg/dl, procalcitonin > 1 μg/l). Steroids were started on day 14 (CSF leucocytes <50 cells/μl) with an initial 5-day pulse of 1 g methylprednisolone. The latter was extended to 10 days after clinical improvement. Following slow steroid tapering over the next 4 weeks, both neurological symptoms and MRI findings substantially ameliorated and the patient –tracheotomized but now vigilant – was transferred to rehabilitation. In a follow-up visit one year later (the patient was in the meantime vaccinated for haemophilus, meningococcus as well as pneumococcus), the patient significantly recovered with residual bilateral hypacusis and mild cerebellar symptoms and was able to walk independently (modified Rankin Scale Score 2). No further relapses occurred. Correspondingly, upon follow-up MRI, the white matter lesions were markedly reduced (Figure 3).

### Discussion

Here we report on a fulminant parainfectious ADEM related to acute pneumococcal meningitis in an unvaccinated splenectomized patient. Some aspects emerge from this instructive example of successful early immunosuppressive treatment in a rare autoimmune mediated parainfectious comorbidity.

First, ADEM is an immune-mediated, monophasic inflammatory disorder of the central nervous system (CNS) and clinical as well as paraclinical differentiation among other primarily demyelinating CNS diseases, especially a first severe relapse of Multiple Sclerosis (MS) often is challenging. Usually, children or younger adults are affected by ADEM but cases of adult onset also have been described (Schwarz et al. 2001; Dale et al. 2001; Tenembaum et al. 2007; Hynson et al. 2001). A monophasic course with a first-time demyelinating inflammatory CNS affection as well as a good response and favorable outcome after steroid therapy is characteristic for ADEM, however, some cases of recurrent or multiphasic courses have also been reported (Tenembaum et al. 2007; Menge et al. 2005). As atypical clinical presentation does not exclude diagnosis of ADEM, analysis of cerebrospinal fluid is important to show mild pleocytosis, elevated protein levels or the transient presence of oligoclonal bands (Menge et al. 2005). Therefore, imaging plays a pivotal role in diagnosing ADEM. Typical non-specific MRI criteria are widespread, multifocal, asymmetric and extensive white matter lesions (typical “periventricular sparing” and lesions of similar inflammatory age) and possible affection of grey matter, with partial contrast enhancement and mainly a restriction in diffusion-weighted sequences (Balasubramanya et al. 2007; Krupp et al. 2007; O’Riordan et al. 1999; Alper 2012; Hu and Lucchinetti 2009). MR-spectroscopy may reveal a reduced N-acetyl-aspartate (NAA) peak as a hint for reversible brain tissue damage, as presented in our case (Bizzi et al. 2001).

Second, these diagnostic criteria also hold true for para-infectious – as well as postvaccinal - ADEM. Infectious diseases precede ADEM-onset in 70-90% of patients, whereas a postvaccinal pathogenesis seems to be rare (Menge et al. 2005; Tenembaum et al. 2007; Schwarz et al. 2001). These preconditions are thought to trigger an overshooting immunologic response leading to ADEM with a characteristic latency of days to weeks, as seen in our case. Upper respiratory tract virus-infections seem to be the most common causes for para-infectious ADEM, whereas Mycoplasma, Borrelia, Chlamydia and Legionella account for the majority of bacterial infections (Okada and Yoshioka 2010; van Assen et al. 2004; Stettner et al. 2012; Heick and Skriver 2000; Spieker et al. 1998; Beleza et al. 2008). With respect to bacterial meningitis, para-infectious ADEM has been related to Haemophilus influenzae and Streptococcus pneumoniae (Beleza et al. 2008; Ohnishi et al. 2007; Jorens et al. 2005; Ueda et al. 2009). In essence, these reports emphasize early diagnosis to initiate corticosteroid treatment (Dale et al. 2009). However, given acute bacterial meningitis and sepsis, debate remains when and in which dosage steroids should be administered. As a key finding we report here that high-dose steroid pulsing appears to be safe and effective (i) when starting not before day 14 after onset of bacterial meningitis, (ii) if CSF leucocytes cell count drops under 50 cells/μl and (iii) despite laboratory values still indicating severe inflammation and sepsis.

Third, the clinical value of brain biopsy is still uncertain given the lack of exact guidelines for the histopathological classification of the disease (Krupp et al. 2007). However, on an individual basis, brain biopsy may be valuable to rule out differential diagnoses such as infection or para-infectious vasculitis, sarcoidosis, neoplasia, metabolic disorders (some of those would require long-term treatment) or disseminated necrotizing leukoencephalopathy (synonyms: Weston-Hurst syndrome, acute necrotizing hemorrhagic encephalomyelitis, ANHEM), which is considered as the severest ADEM variant (Tenembaum et al. 2007; Menge et al. 2005). Brain biopsy in ADEM is usually reported to show non-confluent demyelination restricted to the perivascular area as well as perivenular T-cell infiltration, as also seen here (Menge et al. 2005). Moreover, brain biopsy may also involve the grey matter at the gray-white junction or within the basal ganglia (Wingerchuk 2003; Hynson et al. 2001). Taken together we believe that our case represents a parainfectious ADEM as (i) brain biopsy revealed suitable histopathological findings, (ii) MR findings were in line with established diagnostic criteria and (iii) an infection preceded symptom onset.

## Conclusion

Acute pneumococcal meningitis may be accompanied by immunological parainfectious processes such as ADEM, cerebral vasculitis or acute necrotizing hemorrhagic encephalomyelitis. Despite a growing body of literature regarding ADEM its exact diagnostic have been defined mainly for pediatric patients (Krupp et al. 2007) and differential diagnosis in adults is still sophisticated. Neurocritical care of these patients is challenging as treatment of acute meningitis must be balanced against aggressive immunosuppression for adequate treatment of parainfectious comorbidities. As shown here, brain biopsy may be helpful for exclusion of differential diagnoses and a high-dose corticosteroid-pulse appears safe and effective if initiated not before two weeks after onset of bacterial meningitis and evidence of substantial CSF leucocytes drop - even in still systemically ill patients due to pneumococcal meningitis.

The patient gave written informed consent and agreement to publication of this case report.
